# Clinical features associated with cerebrospinal fluid Epstein–Barr virus positivity in autoimmune GFAP astrocytopathy: a multicenter retrospective cohort study

**DOI:** 10.3389/fneur.2026.1906448

**Published:** 2026-08-27

**Authors:** Xing Mao, Qianqian Qu, Chao Quan, Haidong Lyu, Jingzi Zhangbao, Xuan Wang, Hongmei Tan, Xiang Li

**Affiliations:** 1 Department of Neurology, Jiaozuo People’s Hospital, Jiaozuo, China; 2 Department of Neurology, Huashan Hospital, Fudan University, Shanghai, China; 3 Department of Infectious Diseases, Huashan Hospital Fudan University, Shanghai, China

**Keywords:** autoimmune GFAP astrocytopathy, cerebrospinal fluid, Epstein–Barr virus, metagenomic next-generation sequencing, neuroinflammation, prognosis

## Abstract

**Background:**

Autoimmune glial fibrillary acidic protein astrocytopathy (GFAP-A) is an inflammatory disorder of the central nervous system. The clinical implications of Epstein–Barr virus (EBV) sequences detected in cerebrospinal fluid (CSF) by metagenomic next-generation sequencing (mNGS) remain uncertain.

**Methods:**

This multicenter retrospective cohort study enrolled 60 patients with GFAP-A screened from electronic medical records of two hospitals (Jan 2019–Dec 2025). Patients were stratified into CSF EBV-positive (*n* = 10) and CSF EBV-negative (*n* = 50) groups based on CSF mNGS results. Demographic characteristics, clinical manifestations, laboratory findings, MRI features, treatment, and outcomes were compared. Bonferroni correction was applied to three clinically prioritized endpoints (CSF white blood cell count, altered consciousness, CSF chloride) selected based on prior GFAP-A literature to limit multiplicity bias; the corrected two-sided significance threshold was set at *α* = 0.05/3 ≈ 0.0167. An exploratory Firth penalized multivariable logistic regression model fitted via R (v4.3.1, logistf package) was used to examine the adjusted associations between CSF mNGS-detectable EBV sequences and acute altered consciousness, adjusting for age and sex to mitigate small-sample bias. All other unadjusted baseline comparisons were considered exploratory and were not adjusted for multiplicity. Spearman’s rank correlation was used to assess the relationships of raw EBV mNGS read counts with peak pretreatment modified Rankin Scale (mRS) score and CSF WBC count among EBV-positive patients.

**Results:**

CSF WBC count was higher in the CSF EBV-positive group than in the CSF EBV-negative group [median, 120 (interquartile range, 105–220) vs. 33 (16–97) × 10⁶/L; raw *p* = 0.004; Bonferroni-adjusted *p* = 0.012]. Altered consciousness was more frequent in the EBV-positive group (80.0% vs. 42.0%; crude odds ratio [OR] = 5.52, 95% confidence interval [CI] 1.06–28.71; raw *p* = 0.039), and CSF chloride was numerically lower [111.50 (104.00–117.00) vs. 115.70 (113.00–120.00) mmol/L; raw *p* = 0.048]; however, neither comparison met the Bonferroni-corrected criterion (adjusted *p* = 0.117 and 0.144, respectively). Firth penalized multivariable logistic regression fitted in R was adjusted for age and sex. CSF EBV positivity exhibited a non-significant trend toward higher odds of acute altered consciousness (OR = 4.10, 95% CI: 0.94–24.84, *p* = 0.060). The extremely wide confidence interval indicated high estimation uncertainty limited by the small EBV-positive subgroup (n = 10). Female sex was associated with lower estimated odds of altered consciousness (OR = 0.17, 95% CI: 0.04–0.58, *p* = 0.004), while age showed no independent association (OR = 0.98, 95% CI: 0.95–1.02, *p* = 0.312). No statistically detectable between-group differences were observed in GFAP-IgG characteristics, MRI findings, relapse, or functional outcomes. Among the 10 EBV-positive patients, raw EBV read counts were not statistically correlated with peak pretreatment mRS score (*r*_s_ = 0.118, *p* = 0.745) or CSF WBC count (*r*_s_ = −0.073, *p* = 0.841).

**Conclusion:**

CSF WBC count was the only clinically prioritized comparison that remained statistically significant after Bonferroni correction, suggesting greater CSF pleocytosis and possibly increased intrathecal inflammatory activity in GFAP-A patients with mNGS-detectable CSF EBV. Differences in altered consciousness and CSF chloride remained inconclusive after multiplicity adjustment. Within the EBV-positive subgroup, raw EBV sequencing reads showed no correlation with inflammatory or disability measures. Current findings do not establish CSF EBV positivity as an independent pathogenic or prognostic biomarker for GFAP-A, and the biological origin of the detected EBV sequences remains uncertain.

## Introduction

Autoimmune glial fibrillary acidic protein astrocytopathy (GFAP-A) is an autoimmune inflammatory disorder of the central nervous system (CNS) characterized by GFAP-IgG positivity, particularly in cerebrospinal fluid (CSF), and a heterogeneous spectrum of neurological manifestations. The disease may involve the meninges, brain parenchyma, spinal cord, optic nerves, and peripheral nervous system. Common clinical features include fever, headache, altered consciousness, seizures, visual disturbance, ataxia, limb weakness, bladder or bowel dysfunction, and autonomic dysfunction (1, 2). Although the clinical and neuroimaging characteristics of GFAP-A have been increasingly recognized, its etiology and pathogenesis remain incompletely understood.

Infections, tumors, and immune dysregulation have been proposed as potential triggers or associated conditions of GFAP-A. Reported neoplastic associations include teratoma, prostate cancer, gastrointestinal adenocarcinoma, melanoma, and carcinoid tumors (3). GFAP-A or GFAP-IgG positivity has also been described in temporal association with several viral infections, including herpes simplex virus (4), Epstein–Barr virus (EBV) (5–8), varicella-zoster virus (9), influenza virus (10), severe acute respiratory syndrome coronavirus 2 (11), and dengue virus (12). However, whether these infections directly initiate GFAP-directed autoimmunity, amplify an established inflammatory response, or merely coexist with GFAP-A remains uncertain.

EBV, also known as human herpesvirus 4, is a double-stranded DNA virus that establishes lifelong latency after primary infection. More than 95% of adults worldwide have evidence of previous EBV infection, and viral reactivation may occur under altered immune conditions (13, 14). EBV is associated with several malignancies and immune-mediated disorders and has also been implicated in neurological diseases. Detection of EBV DNA or sequencing reads in CSF may reflect active intrathecal infection or reactivation, carriage within EBV-latent B cells, contamination from the peripheral circulation, or low-level technical background. Therefore, the presence of EBV sequences in CSF should not, by itself, be considered definitive evidence of EBV encephalitis or a causal relationship with concurrent neurological disease.

Several case reports and small case series have described GFAP-A accompanied by CSF EBV positivity (5–8). More recently, intrathecal EBV reactivation has been reported in patients with GFAP-A, raising the possibility that EBV may be associated with the inflammatory phenotype of the disease (15). Nevertheless, systematic comparisons between GFAP-A patients with and without CSF EBV detection remain limited. In particular, it is unclear whether CSF EBV positivity is associated with greater CSF inflammatory activity, distinctive clinical or neuroimaging features, or different long-term outcomes. The interpretation of metagenomic next-generation sequencing (mNGS) findings is further complicated by the high prevalence of latent EBV infection and the semi-quantitative nature of raw sequencing read counts.

In this multicenter retrospective cohort study, we compared the demographic characteristics, clinical manifestations, laboratory findings, MRI features, treatment patterns, and outcomes of patients with GFAP-A according to CSF EBV status determined by mNGS. We also explored whether raw EBV mNGS read counts were associated with CSF inflammatory burden or acute neurological disability among EBV-positive patients. The study was intended to characterize the clinical correlates of CSF EBV detection in GFAP-A rather than to establish a causal relationship between EBV and GFAP-A.

## Methods

### Study design and participants

**Figure 1:**
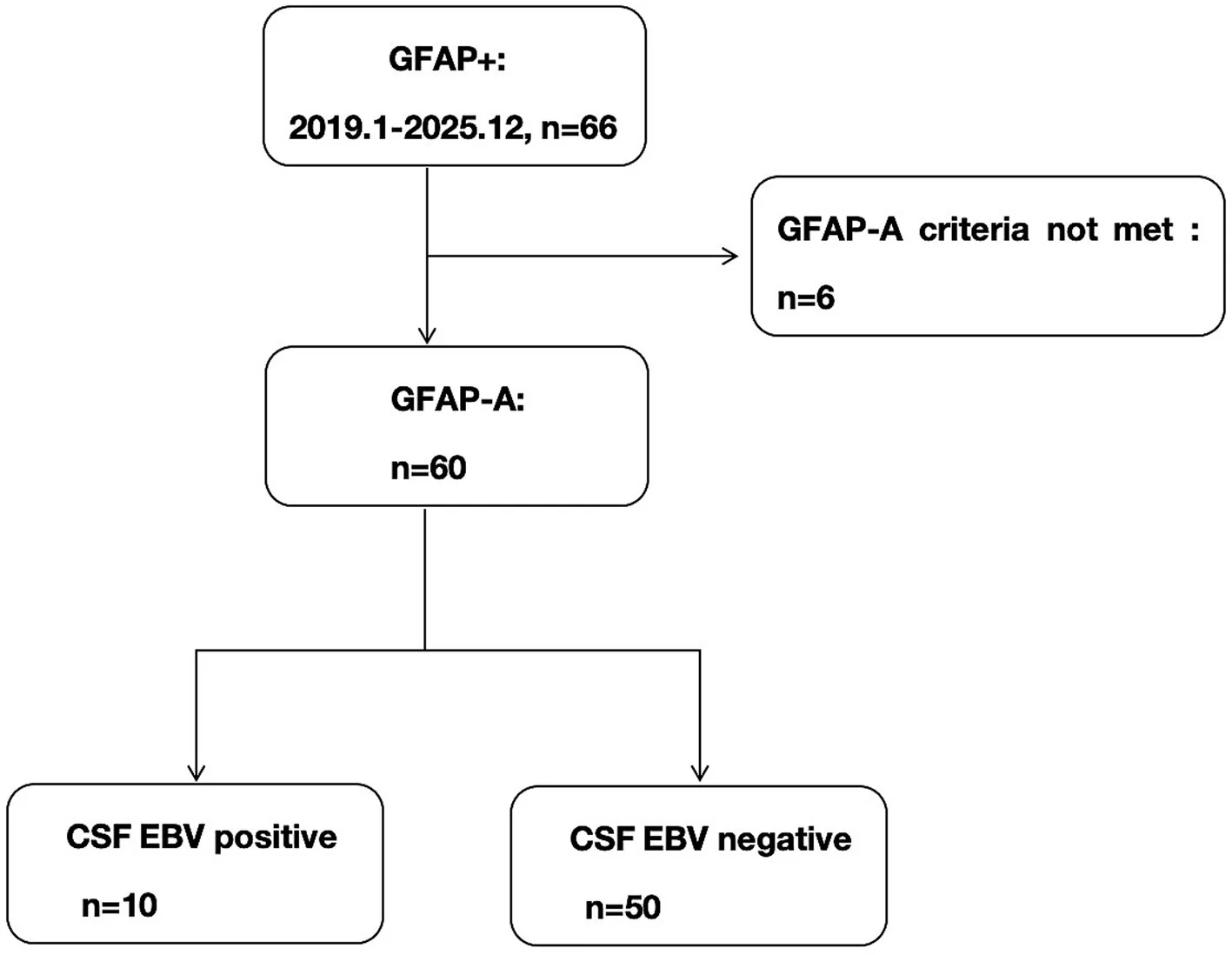
Flowchart of patient screening, enrollment, and group assignment. A total of 66 patients with serum and/or CSF GFAP-IgG positivity were screened at the two participating centers. After application of the eligibility criteria, 60 patients with autoimmune GFAP astrocytopathy were included and classified according to CSF mNGS findings into the CSF EBV-positive group (*n* = 10) and CSF EBV-negative group (*n* = 50). CSF, cerebrospinal fluid; EBV, Epstein–Barr virus; GFAP-IgG, glial fibrillary acidic protein immunoglobulin G; mNGS, metagenomic next-generation sequencing.

This multicenter retrospective cohort study identified patients by retrieving electronic medical records of individuals admitted to the Department of Neurology at Huashan Hospital, Fudan University, and Jiaozuo People’s Hospital between January 2019 and December 2025. We initially screened 66 patients with serum and/or cerebrospinal fluid (CSF) GFAP-IgG positivity. Following screening against predefined eligibility criteria, 60 patients were retained for the final analysis (Figure 1).

The inclusion criteria were as follows:

1. Acute or subacute onset of encephalitis, meningoencephalitis, myelitis, or a combination of these syndromes.
2. GFAP-IgG positivity in CSF, with or without concurrent serum GFAP-IgG positivity.
3. Clinical, laboratory, and neuroimaging findings compatible with autoimmune GFAP astrocytopathy (GFAP-A).
4. Availability of CSF metagenomic next-generation sequencing (mNGS) results for determining EBV status.

Patients were excluded if their neurological syndrome was better explained by an alternative diagnosis, including central nervous system (CNS) malignancy, lymphoma, tuberculosis, another confirmed CNS infection, or another neurological disorder. No patient with isolated serum GFAP-IgG positivity was included.

Patients were classified according to CSF mNGS EBV detection status. CSF EBV positivity was defined as an EBV raw sequencing read count of at least 30 together with EBV genome coverage of at least 5%. These thresholds were predefined according to the laboratory interpretation criteria used at the participating centers. Patients meeting both criteria were assigned to the CSF EBV-positive group, whereas those who did not meet these criteria were assigned to the CSF EBV-negative group.

Demographic, clinical, laboratory, neuroimaging, treatment, and follow-up data were retrospectively extracted from electronic medical records using a standardized data collection form. Demographic variables included age and sex. Clinical variables included fever, headache, meningeal signs, altered consciousness, limb weakness, bladder or bowel dysfunction, seizures, and respiratory failure.

Peripheral blood measurements included white blood cell (WBC) count, C-reactive protein (CRP), and erythrocyte sedimentation rate (ESR). CSF measurements included opening pressure, WBC count, protein, glucose, chloride, cytology, and oligoclonal bands (OCBs).

All laboratory biomarkers (serum and cerebrospinal fluid) as well as CSF mNGS specimens were obtained from the same lumbar puncture performed before initiation of immunotherapy and anti-infective therapy (including acyclovir).

Serum and CSF GFAP-IgG were assessed using cell-based assay (CBA) and tissue-based assay (TBA). HEK293 cells expressing GFAP antigen were used as the substrate for CBA, and frozen sections of normal human brain tissue were used for TBA. Both assays were performed using indirect immunofluorescence. GFAP-IgG positivity was determined according to the presence of a characteristic astrocytic immunofluorescence pattern.

All immunofluorescence results were independently reviewed by two experienced neuroimmunologists who were blinded to CSF EBV status, and agreement between the two reviewers was required for the final interpretation. Antibody titers were determined by serial dilution when quantitative results were available. Some patients had only qualitative TBA-positive results without reportable serial-dilution titers.

GFAP-IgG status was categorized as isolated CSF positivity, defined as CSF GFAP-IgG positivity with a negative serum result, or dual serum–CSF positivity, defined as positivity in both specimens. Quantitative CSF GFAP-IgG titers and the frequency of qualitative TBA-only positivity were compared between the EBV-status groups in exploratory analyses (Supplementary Table S1).

All CSF samples were processed within 2 h post-collection following identical unified mNGS workflows across both study centers to minimize inter-center technical heterogeneity. Blank negative controls were included in each sequencing batch to monitor potential exogenous contamination. The criteria for CSF EBV positivity were an EBV raw read count of at least 30 and genome coverage of at least 5%.

Raw EBV sequencing read counts were retained for an exploratory semi-quantitative analysis among patients with CSF mNGS-detectable EBV. These raw counts were not normalized for total sequencing depth and were not considered equivalent to quantitative viral loads measured using calibrated polymerase chain reaction (PCR).

Peripheral blood EBV DNA PCR was performed in all 10 CSF EBV-positive patients during the same clinical episode and was negative in every patient (Supplementary Table S2). Complete EBV serological panels, including viral capsid antigen IgM and IgG, early antigen IgG, and EBV nuclear antigen IgG, were not consistently available because of the retrospective study design and limitations in stored biospecimens. Orthogonal confirmation of CSF EBV using quantitative PCR or digital droplet PCR and serial post-treatment CSF mNGS testing were not routinely performed.

All patients underwent both brain and spinal MRI before treatment. Brain MRI protocols included T1-weighted, T2-weighted, fluid-attenuated inversion recovery, diffusion-weighted, apparent diffusion coefficient, and contrast-enhanced sequences. Spinal MRI included T1-weighted, T2-weighted, and contrast-enhanced sequences.

MRI findings were reviewed for brain parenchymal involvement, periventricular radial linear enhancement, meningeal involvement, spinal cord involvement, and spinal leptomeningeal involvement. Because brain and spinal MRI results were available for all included patients, the denominators used for imaging comparisons were 10 and 50 for the CSF EBV-positive and CSF EBV-negative groups, respectively.

Treatment decisions were made by the attending physicians according to clinical severity and routine practice at the participating centers. Acute-phase immunotherapy included intravenous methylprednisolone (IVMP), intravenous immunoglobulin (IVIG), plasma exchange, or combinations of these treatments. IVMP monotherapy was defined as acute treatment with IVMP without concurrent IVIG or plasma exchange. Maintenance treatment included oral corticosteroids, with or without additional immunosuppressive therapy such as mycophenolate mofetil, rituximab, or azathioprine.

All 10 CSF EBV-positive patients received intravenous acyclovir at a dose of 10 mg/kg every 8 h for 14–21 days. Antiviral treatment was initiated within 72 h after the diagnosis of GFAP-A. Because antiviral treatment was allocated according to CSF EBV status rather than through random assignment, its independent association with clinical outcomes could not be estimated.

Follow-up information was obtained through outpatient visits and structured telephone interviews. Neurological disability was assessed using the modified Rankin Scale (mRS). Peak pretreatment mRS was defined as the highest mRS score recorded before the initiation of immunotherapy. Functional status was reassessed at the last available follow-up, and an mRS score of 2 or lower was defined as a favorable outcome.

Relapse was defined as the development of new or worsening neurological deficits lasting at least 24 h, occurring more than 1 month after treatment initiation, and not attributable to fever, infection, metabolic disturbance, or another systemic trigger. Follow-up duration was calculated from the index admission to the last available clinical assessment.

### Statistical analysis

Statistical analyses were performed using SPSS version 29.0. Age and follow-up duration are presented as mean ± standard deviation and were compared using the independent-samples t test. Other continuous or ordinal variables are presented as median and interquartile range and were compared using the Mann–Whitney U test. Categorical variables are presented as number and percentage and were compared using Fisher’s exact test. Crude odds ratios (ORs) with 95% confidence intervals (CIs) were calculated for categorical comparisons using the CSF EBV-negative group as the reference. All tests were two-sided.

To account for multiple testing, Bonferroni correction was applied to three clinically prioritized comparisons (CSF WBC count, altered consciousness, and CSF chloride concentration). These three endpoints were selected based on previously reported associations with disease severity and inflammatory phenotypes in GFAP-A (16, 17) as core markers of intrathecal inflammatory severity and clinical encephalopathy phenotype. Both raw and Bonferroni-adjusted *p* values are reported for these comparisons. Because this adjustment was not part of a formal pre-specified statistical analysis plan, the results from the adjusted comparisons should be interpreted as hypothesis-generating rather than confirmatory. All other demographic, clinical, laboratory, MRI, treatment, and outcome comparisons were considered exploratory and were not adjusted for multiplicity.

To reduce small-sample and separation bias caused by only 10 CSF EBV-positive patients, we performed Firth penalized multivariable logistic regression via R (version 4.3.1, logistf package) as a sensitivity analysis. The binary outcome was acute altered consciousness at onset, with CSF EBV positivity as the primary predictor (CSF EBV-negative as reference), adjusted for continuous age and binary sex. We reported regression coefficients (*β*), odds ratios (OR), profile-likelihood 95% confidence intervals (CIs), and two-sided penalized *p* values. Spearman’s rank correlation was used to explore the associations of raw EBV mNGS read counts with peak pretreatment mRS score and CSF WBC count. Given the small number of CSF EBV-positive patients, the regression and correlation analyses were considered exploratory and were interpreted cautiously.

Missing data were handled using complete-case analysis. No data were missing for the three principal comparisons or for the MRI variables included in the main analyses.

## Results

A total of 66 patients with serum and/or CSF GFAP-IgG positivity were screened at the two participating centers. After application of the eligibility criteria, 60 patients with GFAP-A were included in the final analysis. According to CSF mNGS findings, 10 patients were classified as CSF EBV-positive and 50 as CSF EBV-negative (Figure 1).

The mean age was 46.10 ± 11.96 years in the CSF EBV-positive group and 47.28 ± 16.45 years in the CSF EBV-negative group (mean difference, −1.18 years; 95% CI, −12.16 to 9.80; *p* = 0.830). Male patients accounted for 80.0% (8/10) and 66.0% (33/50) of the two groups, respectively (crude OR = 2.06, 95% CI 0.39–10.80; *p* = 0.480) (Table 1).

**Table 1:** Demographic and clinical characteristics of patients with GFAP-A according to CSF EBV status.

Variable	CSF EBV-positive group (*n* = 10)	CSF EBV-negative group (*n* = 50)	Effect estimate (95% CI)	Raw *p*-value	Bonferroni-adjusted *p*-value
Male sex, *n* (%)	8 (80.0)	33 (66.0)	OR, 2.06 (0.39–10.80)	0.48	—
Age, years, mean ± SD	46.10 ± 11.96	47.28 ± 16.45	Mean difference, −1.18 (−12.16 to 9.80)	0.83	—
Clinical features					
Fever, *n* (%)	10 (100.0)	35 (70.0)	Not estimable	0.054	—
Headache, *n* (%)	8 (80.0)	33 (66.0)	OR, 2.06 (0.39–10.80)	0.48	—
Meningeal signs, *n* (%)	9 (90.0)	30 (60.0)	OR, 6.00 (0.70–51.10)	0.084	—
Altered consciousness, *n* (%)	8 (80.0)	21 (42.0)	OR, 5.52 (1.06–28.71)	0.039	0.117
Limb weakness, *n* (%)	7 (70.0)	33 (66.0)	OR, 1.20 (0.28–5.25)	1	—
Bladder/bowel dysfunction, *n* (%)	6 (60.0)	30 (60.0)	OR, 1.00 (0.25–4.00)	1	—
Seizures, *n* (%)	2 (20.0)	8 (16.0)	OR, 1.31 (0.23–7.36)	0.668	—
Respiratory failure, *n* (%)	3 (30.0)	7 (14.0)	OR, 2.63 (0.55–12.66)	0.347	—

Data are presented as mean ± standard deviation or number (percentage). Age was compared using the independent-samples t test, and categorical variables were compared using Fisher’s exact test. Crude odds ratios were calculated with the CSF EBV-negative group as the reference. The odds ratio for fever was not estimated because no patient in the CSF EBV-positive group was without fever. Raw two-sided P values are reported for all comparisons. Bonferroni correction was applied only to the three clinically prioritized comparisons (altered consciousness, CSF WBC count, and CSF chloride concentration), and adjusted p values were calculated as min (3 × raw P, 1.000). The remaining comparisons were exploratory and were not adjusted for multiplicity.

CI, confidence interval; CSF, cerebrospinal fluid; EBV, Epstein–Barr virus; GFAP-A, autoimmune glial fibrillary acidic protein astrocytopathy; OR, odds ratio; SD, standard deviation.

Altered consciousness was observed in 80.0% (8/10) of the CSF EBV-positive group and 42.0% (21/50) of the CSF EBV-negative group (crude OR = 5.52, 95% CI 1.06–28.71; raw *p* = 0.039). However, this comparison did not meet the Bonferroni-corrected significance criterion (adjusted *p* = 0.117).

Fever was present in all 10 CSF EBV-positive patients and in 35 of 50 CSF EBV-negative patients (100.0% vs. 70.0%; *p* = 0.054). Meningeal signs were observed in 90.0% and 60.0% of patients, respectively (crude OR = 6.00, 95% CI 0.70–51.10; *p* = 0.084). These were exploratory comparisons, and their effect estimates were imprecise. No statistically detectable between-group differences were observed for headache, limb weakness, bladder/bowel dysfunction, seizures, or respiratory failure (Table 1). Individual clinical characteristics of the 10 CSF EBV-positive patients are presented in Supplementary Table S2.

Peripheral blood WBC count was numerically higher in the CSF EBV-positive group than in the CSF EBV-negative group [median, 8.46 (IQR, 6.50–9.52) vs. 6.39 (5.24–8.28) × 10^9^/L; *p* = 0.065]. ESR and CRP did not show statistically detectable between-group differences in exploratory analyses (Table 2).

**Table 2:** Laboratory and MRI findings of patients with GFAP-A according to CSF EBV status.

Variable	CSF EBV-positive group (*n* = 10)	CSF EBV-negative group (*n* = 50)	Effect estimate (95% CI)	Raw *p*-value	Bonferroni-adjusted *p*-value
Peripheral blood parameters					
WBC, ×10^9^/L	8.46 (6.50, 9.52)	6.39 (5.24, 8.28)	—	0.065	—
ESR, mm/h	10.50 (9.00, 14.00)	10.00 (5.00, 18.00)	—	0.758	—
CRP, mg/L	0.87 (0.50, 1.63)	1.43 (0.50, 4.45)	—	0.191	—
CSF parameters					
Opening pressure, mmH₂O	215.00 (150.00, 260.00)	165.00 (120.00, 240.00)	—	0.266	—
WBC, ×10⁶/L	120.00 (105.00, 220.00)	33.00 (16.00, 97.00)	—	0.004	0.012
Protein, g/L	1.47 (1.00, 1.79)	1.24 (0.71, 1.94)	—	0.242	—
Glucose, mmol/L	2.59 (2.38, 2.90)	2.70 (2.44, 3.23)	—	0.168	—
Chloride, mmol/L	111.50 (104.00, 117.00)	115.70 (113.00, 120.00)	—	0.048	0.144
Brain MRI findings					
Brain parenchymal involvement, *n* (%)	9 (90.0)	33 (66.0)	4.64 (0.54–39.69)	0.256	—
Periventricular radial linear enhancement, *n* (%)	2 (20.0)	15 (30.0)	0.58 (0.11–3.09)	0.709	—
Meningeal involvement, *n* (%)	6 (60.0)	30 (60.0)	1.00 (0.25–4.00)	1	—
Spinal MRI findings					
Spinal cord involvement, *n* (%)	6 (60.0)	23 (46.0)	1.76 (0.44–7.01)	0.5	—
Spinal leptomeningeal involvement, *n* (%)	8 (80.0)	31 (62.0)	2.45 (0.47–12.78)	0.47	—

Continuous variables are presented as median (interquartile range) and were compared using the Mann–Whitney U test. Categorical variables are presented as number (percentage) and were compared using Fisher’s exact test. Crude odds ratios with 95% confidence intervals were calculated for categorical variables using the CSF EBV-negative group as the reference. All patients underwent both brain and spinal MRI; therefore, the denominators for the MRI comparisons were 10 and 50, respectively. Raw two-sided P values are reported for all comparisons. Bonferroni correction was applied only to the three clinically prioritized comparisons: altered consciousness, CSF WBC count, and CSF chloride concentration. Bonferroni-adjusted p values were calculated as min (3 × raw P, 1.000). The adjusted p value for altered consciousness is reported in Table 1. All remaining comparisons were exploratory and were not adjusted for multiplicity.

CI, confidence interval; CRP, C-reactive protein; CSF, cerebrospinal fluid; EBV, Epstein–Barr virus; ESR, erythrocyte sedimentation rate; GFAP-A, autoimmune glial fibrillary acidic protein astrocytopathy; MRI, magnetic resonance imaging; OR, odds ratio; WBC, white blood cell.

Among the three clinically prioritized comparisons, CSF WBC count was higher in the CSF EBV-positive group than in the CSF EBV-negative group [120.00 (105.00–220.00) vs. 33.00 (16.00–97.00) × 10⁶/L; raw *p* = 0.004]. This was the only comparison that remained statistically significant after Bonferroni correction (adjusted *p* = 0.012).

CSF chloride concentration was numerically lower in the CSF EBV-positive group [111.50 (104.00–117.00) vs. 115.70 (113.00–120.00) mmol/L; raw *p* = 0.048], but the difference did not meet the Bonferroni-corrected significance criterion (adjusted *p* = 0.144). No statistically detectable differences were observed in CSF opening pressure, protein, or glucose in the exploratory comparisons (Table 2).

No patient with isolated serum GFAP-IgG positivity was included. Isolated CSF GFAP-IgG positivity was present in 60.0% (6/10) of CSF EBV-positive patients and 64.0% (32/50) of CSF EBV-negative patients. Dual serum–CSF GFAP-IgG positivity was present in 40.0% (4/10) and 36.0% (18/50), respectively. Neither distribution differed statistically between the groups (both *p* = 1.000) (Supplementary Table S1).

Among patients with available serial-dilution titers, the median CSF GFAP-IgG titer was 1:100 (IQR, 1:49–1:100; *n* = 8) in the CSF EBV-positive group and 1:32 (IQR, 1:10–1:100; *n* = 42) in the CSF EBV-negative group (*p* = 0.147). Qualitative TBA-only CSF positivity was observed in 20.0% (2/10) and 16.0% (8/50) of patients, respectively (*p* = 0.668) (Supplementary Table S1).

Among the 10 CSF EBV-positive patients, raw EBV mNGS read counts ranged from 30 to 1,087. Peripheral blood EBV DNA PCR was negative in all 10 patients (Supplementary Table S2). In exploratory Spearman analyses, no statistically detectable monotonic association was observed between raw EBV read counts and either peak pretreatment mRS score (Spearman’s r_s_ = 0.118, *p* = 0.745) or CSF WBC count (r_s_ = −0.073, *p* = 0.841).

All patients underwent both brain and spinal MRI. Brain parenchymal involvement was identified in 90.0% (9/10) of the CSF EBV-positive group and 66.0% (33/50) of the CSF EBV-negative group (crude OR = 4.64, 95% CI 0.54–39.69; *p* = 0.256). Periventricular radial linear enhancement was observed in 20.0% and 30.0%, respectively (*p* = 0.709), while meningeal involvement was present in 60.0% of both groups (*p* = 1.000).

**Figure 2:**
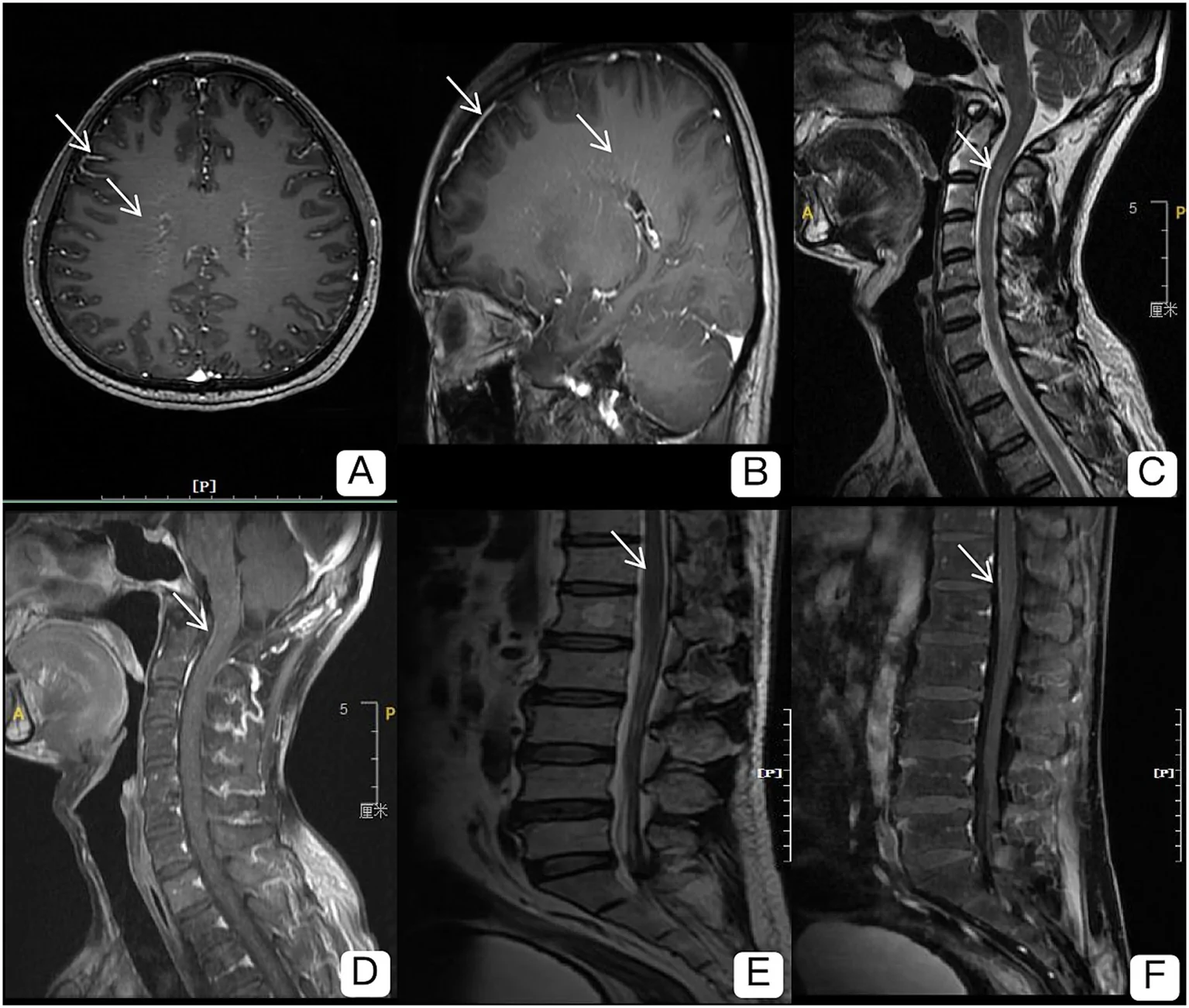
Representative brain and spinal MRI findings in patients with autoimmune GFAP astrocytopathy and CSF EBV positivity. Contrast-enhanced axial brain MRI shows characteristic periventricular radial linear enhancement **(A,B)**. Sagittal cervical spinal cord t2-weighted and contrast-enhanced t1-weighted images demonstrate intramedullary signal abnormalities and leptomeningeal enhancement **(C,D)**. Sagittal lumbar spinal MRI demonstrates spinal leptomeningeal and parenchymal inflammatory abnormalities **(E,F)**. CSF, cerebrospinal fluid; EBV, Epstein–Barr virus; GFAP, glial fibrillary acidic protein; MRI, magnetic resonance imaging.

Spinal cord involvement was observed in 60.0% (6/10) of CSF EBV-positive patients and 46.0% (23/50) of CSF EBV-negative patients (crude OR = 1.76, 95% CI 0.44–7.01; *p* = 0.500). Spinal leptomeningeal involvement was present in 80.0% (8/10) and 62.0% (31/50), respectively (crude OR = 2.45, 95% CI 0.47–12.78; *p* = 0.470). Thus, no statistically detectable between-group differences were observed for the assessed MRI findings, although the confidence intervals were wide (Table 2). Representative brain and spinal MRI findings in CSF EBV-positive patients are shown in Figure 2.

Both groups received immunotherapy-centered treatment. IVMP monotherapy during the acute phase was administered to 40.0% (4/10) of the CSF EBV-positive group and 58.0% (29/50) of the CSF EBV-negative group (crude OR = 0.48, 95% CI 0.12–1.93; *p* = 0.322). Oral corticosteroids were used during maintenance treatment in 80.0% (8/10) and 68.0% (34/50), respectively (crude OR = 1.88, 95% CI 0.36–9.90; *p* = 0.708). All 10 CSF EBV-positive patients received intravenous acyclovir (Table 3 and Supplementary Table S2).

**Table 3:** Treatment and outcomes of patients with GFAP-A according to CSF EBV status.

Variable	CSF EBV-positive group (*n* = 10)	CSF EBV-negative group (*n* = 50)	Crude OR (95% CI)	Raw *p*-value
Treatment				
IVMP monotherapy during the acute phase, *n* (%)	4 (40.0)	29 (58.0)	0.48 (0.12–1.93)	0.322
Oral corticosteroids during maintenance, *n* (%)	8 (80.0)	34 (68.0)	1.88 (0.36–9.90)	0.708
Functional outcomes				
Peak pretreatment mRS score	4.50 (3.75, 5.00)	4.00 (3.00, 4.00)	—	0.156
mRS score at last follow-up	1.50 (1.00, 3.00)	2.00 (1.00, 3.00)	—	0.582
Favorable outcome at last follow-up (mRS ≤ 2), *n* (%)	7 (70.0)	36 (72.0)	0.91 (0.21–4.01)	1
Relapse during follow-up, *n* (%)	1 (10.0)	9 (18.0)	0.51 (0.06–4.52)	1
Follow-up duration, months, mean ± SD	30.80 ± 14.52	23.82 ± 15.07	—	0.184

Continuous and ordinal variables are presented as median (interquartile range), except for follow-up duration, which is presented as mean ± standard deviation. Peak pretreatment and last follow-up mRS scores were compared using the Mann–Whitney U test, and follow-up duration was compared using the independent-samples t test. Categorical variables are presented as number (percentage) and were compared using Fisher’s exact test. Crude odds ratios with 95% confidence intervals were calculated using the CSF EBV-negative group as the reference. All p values are raw two-sided values. Treatment and outcome comparisons were exploratory and were not adjusted for multiplicity. All patients in the CSF EBV-positive group received intravenous acyclovir; because antiviral treatment was assigned according to CSF EBV status rather than randomly, no comparative effect estimate was calculated for antiviral therapy.

CI, confidence interval; CSF, cerebrospinal fluid; EBV, Epstein–Barr virus; IVMP, intravenous methylprednisolone; mRS, modified Rankin Scale; OR, odds ratio; SD, standard deviation.

The median peak pretreatment mRS score was 4.50 (IQR, 3.75–5.00) in the CSF EBV-positive group and 4.00 (IQR, 3.00–4.00) in the CSF EBV-negative group (*p* = 0.156). At the last follow-up, the median mRS scores were 1.50 (IQR, 1.00–3.00) and 2.00 (IQR, 1.00–3.00), respectively (*p* = 0.582).

A favorable outcome at the last follow-up was observed in 70.0% (7/10) of CSF EBV-positive patients and 72.0% (36/50) of CSF EBV-negative patients (crude OR = 0.91, 95% CI 0.21–4.01; *p* = 1.000). Relapse occurred in 10.0% (1/10) and 18.0% (9/50), respectively (crude OR = 0.51, 95% CI 0.06–4.52; *p* = 1.000). The mean follow-up duration was 30.80 ± 14.52 months in the CSF EBV-positive group and 23.82 ± 15.07 months in the CSF EBV-negative group (*p* = 0.184) (Table 3). These exploratory outcome comparisons did not identify statistically detectable differences between the groups, but the estimates were imprecise because of the small CSF EBV-positive subgroup.

We constructed a Firth penalized multivariable logistic regression model in R’s logistf package to explore factors associated with acute altered consciousness, adjusting for age and sex (Table 4). CSF EBV positivity was numerically associated with elevated odds of altered consciousness but did not reach statistical significance (*β* = 1.41, OR = 4.10, 95% CI: 0.94–24.84, *p* = 0.0603). The confidence interval crossed the null value of 1.0, and the broad interval range reflected severe estimation uncertainty due to only 10 CSF EBV-positive patients.

**Table 4:** Exploratory Firth penalized logistic regression of factors associated with acute altered consciousness.

Predictor	*β*	Adjusted OR	95% CI	*p*-value
Intercept	1.10	3.00	0.46–23.80	0.257
CSF EBV positivity	1.41	4.10	0.94–24.84	0.060
Age, per 1-year increase	−0.02	0.98	0.95–1.02	0.312
Sex (female vs. male)	−1.78	0.17	0.04–0.58	0.004

Firth penalized multivariable logistic regression was conducted in R v4.3.1 with the logistf package to resolve sparse-data separation bias among 10 CSF EBV-positive subjects. CSF EBV-negative patients and male patients were set as reference groups for binary predictors. Profile-likelihood-based 95% confidence intervals (CIs) were derived from regression models. All estimates are exploratory and should be interpreted cautiously because of the small sample size. The wide confidence interval for CSF EBV positivity crosses the null value of 1.0, indicating a non-significant trend with high estimation uncertainty.

CSF, cerebrospinal fluid; EBV, Epstein–Barr virus; *β*, regression coefficient; OR, odds ratio; CI, confidence interval.

Age showed no statistically significant association with altered consciousness after adjustment (*β* = −0.02, OR = 0.98, 95% CI: 0.95–1.02, *p* = 0.312). Relative to male sex, female sex was associated with lower odds of acute consciousness disturbance (*β* = −1.78, OR = 0.167, 95% CI: 0.04–0.58, *p* = 0.004). This inverse association between female sex and altered consciousness remained present after further adjustment for age and CSF EBV status. The model intercept (*β* = 1.10, OR = 3.00, 95% CI: 0.46–23.80, *p* = 0.257) represents only a baseline constant with no clinical interpretive value.

## Discussion

In this multicenter retrospective cohort, we compared the clinical characteristics of patients with GFAP-A according to the presence or absence of EBV sequences detected by CSF mNGS. The principal finding was that CSF WBC count was higher in the CSF EBV-positive group and was the only clinically prioritized comparison that remained statistically significant after Bonferroni correction. Altered consciousness was more frequent and CSF chloride concentration was lower in the EBV-positive group in the unadjusted analyses, but neither difference met the corrected significance criterion. We did not identify statistically detectable differences in GFAP-IgG profiles, MRI findings, relapse, or functional outcomes. Within the EBV-positive subgroup, raw EBV sequencing read counts were not statistically correlated with CSF WBC count or peak pretreatment mRS score.

Several reports have described EBV detection in patients with GFAP-A or GFAP-IgG-positive neurological syndromes (5–8). EBV-related CNS disorders and GFAP-A may share clinical features, including fever, headache, meningeal signs, altered consciousness, myelitis, and CSF pleocytosis (18, 19). This overlap makes it difficult to determine whether EBV represents a direct CNS infection, an immunological trigger, secondary reactivation during autoimmune inflammation, or an incidental laboratory finding. The higher CSF WBC count observed in the EBV-positive group suggests that detectable CSF EBV sequences may occur in the setting of greater CSF inflammatory activity. However, the present observational association does not establish that EBV initiated or aggravated the inflammatory response. Increased migration of EBV-latent B cells into the CSF during active autoimmune inflammation could provide an alternative explanation for the mNGS signal. This possibility is consistent with the absence of peripheral blood EBV DNAemia in our cohort. Critically, the three endpoints selected for Bonferroni multiplicity adjustment were chosen based on prior GFAP-A literature (16, 17), rather than selected *post hoc* after observing unadjusted *p* values. This literature-guided selection reduces the risk of selective reporting of significant findings and strengthens the reliability of the statistically significant intergroup difference in CSF WBC count after correction.

Several biological mechanisms could potentially link EBV to CNS autoimmunity. EBV establishes latency primarily in B cells and may influence B-cell activation, antigen presentation, and inflammatory signaling (13, 14). Experimental studies have also suggested that EBV may interact with glial cells and induce inflammatory responses (20, 21). Molecular mimicry between EBV antigens and host CNS proteins has been demonstrated in other autoimmune neurological diseases (22, 23). These observations provide biological plausibility for an association between EBV and neuroinflammation, but they do not demonstrate an EBV-specific pathogenic mechanism in GFAP-A. Because the present study did not include cellular localization of EBV, intrathecal antibody indices, or mechanistic experiments, the biological origin and pathogenic significance of the detected viral sequences remain uncertain.

Altered consciousness was more frequent among EBV-positive patients, and both the crude and age- and sex-adjusted ORs showed a numerically increased but statistically uncertain association. Nevertheless, the Bonferroni-adjusted group comparison did not reach statistical significance, and the confidence interval from the exploratory regression model was wide and included the null. The finding may reflect an association that this small study was insufficiently powered to confirm, but random variation and residual confounding cannot be excluded. Altered consciousness may be influenced by several factors, including the extent of CNS inflammation, seizures, respiratory failure, metabolic abnormalities, overlapping neuronal autoantibodies, and treatment timing. Standardized testing for neuronal surface antibodies was not performed in every participant, which further limits interpretation. Accordingly, these results should be considered hypothesis-generating rather than evidence that CSF EBV positivity independently predicts encephalopathy severity.

Female sex showed an inverse association with altered consciousness in the exploratory model (OR = 0.17, 95% CI 0.04–0.58, *p* = 0.004); however, this finding was not a prespecified hypothesis and may represent an unstable estimate caused by the limited sample size. This was not a principal study question, and the estimate was obtained from a small model with limited numbers in several categories. It may therefore be unstable or affected by unmeasured differences in disease phenotype. This observation requires independent confirmation and should not currently be regarded as an established sex-related effect in GFAP-A.

CSF chloride concentration was numerically lower in the EBV-positive group, but the difference did not meet the Bonferroni-corrected criterion. CSF chloride is a nonspecific measure that may be affected by systemic electrolyte status, hydration, nutrition, and other concurrent conditions. In the absence of a statistically robust association or a clearly supported biological mechanism, this finding remains inconclusive. By contrast, CSF WBC count represented the most consistent between-group difference in this cohort. Nevertheless, the distributions overlapped between groups, and CSF pleocytosis alone cannot distinguish EBV-related CNS infection or reactivation from GFAP-A-associated autoimmune inflammation.

GFAP-IgG positivity patterns and quantitative CSF titers did not show statistically detectable differences according to EBV status. These findings do not suggest a clearly distinct GFAP-IgG profile in patients with detectable CSF EBV. However, serial-dilution titers were not available for every patient, and some results were based only on qualitative TBA positivity. Longitudinal antibody measurements were also unavailable. A relationship between EBV activity and the development or persistence of GFAP-directed immune responses therefore cannot be excluded.

No statistically detectable differences were observed in the assessed brain or spinal MRI findings. Characteristic periventricular radial linear enhancement was present in both groups, and no clear differences were identified in brain parenchymal, meningeal, spinal cord, or spinal leptomeningeal involvement. These findings suggest that CSF EBV-positive patients did not demonstrate an obviously distinct neuroimaging pattern in this cohort. However, the effect estimates were imprecise, and the absence of statistical significance should not be interpreted as evidence that the imaging patterns were equivalent.

Raw EBV mNGS read counts were not statistically correlated with either CSF WBC count or peak pretreatment mRS score among the 10 EBV-positive patients. This differs from reports suggesting an association between quantitative intrathecal EBV load and neurological severity in GFAP-A (15). The discrepancy may partly reflect methodological differences. Raw mNGS read counts are influenced by sequencing depth, sample composition, nucleic acid extraction, and bioinformatic processing and are not equivalent to viral copy numbers measured by calibrated quantitative PCR. In addition, the correlation analyses were restricted to 10 patients who had already met the EBV-positive threshold, resulting in limited statistical power and a restricted range of values. Therefore, the absence of statistically detectable correlations does not exclude a dose–response relationship or establish that EBV abundance has no clinical relevance.

Peripheral blood EBV DNA PCR was negative in all EBV-positive patients, which does not support substantial systemic EBV DNAemia at the time of testing. However, this finding cannot establish that the detected viral signal represented localized CNS reactivation. Complete EBV serological profiles, quantitative CSF EBV PCR or digital droplet PCR, intrathecal EBV antibody indices, and serial post-treatment CSF testing were unavailable. Although batch-level negative controls and predefined mNGS thresholds were used, low-level analytical background cannot be entirely excluded, particularly for cases close to the positivity threshold. In this study, “CSF EBV-positive” should therefore be understood as meeting the specified mNGS criteria rather than as a definitive diagnosis of EBV encephalitis.

We did not identify statistically detectable differences in relapse or functional outcomes between the groups. These results differ from observations in cohorts of viral encephalitis in which neuronal or glial autoantibody positivity was associated with poorer outcomes (24). However, the study populations are not directly comparable: our cohort consisted of patients with established GFAP-A, whereas the previous study evaluated autoimmunity occurring in the setting of viral encephalitis. Treatment differences are also important. All EBV-positive patients in the present cohort received acyclovir in addition to immunotherapy, and antiviral treatment was assigned according to EBV status rather than randomly. Consequently, the observed outcomes reflect the combined treatment approach used in clinical practice and cannot separate the effects of EBV status, antiviral therapy, and immunotherapy. In addition, the small number of relapses and variation in follow-up duration limited assessment of cumulative relapse risk.

The findings should be interpreted in the context of the retrospective design and the small EBV-positive subgroup. The limited sample size resulted in wide confidence intervals and reduced the ability to detect clinically relevant differences, particularly in the regression and correlation analyses. Although Bonferroni correction was applied to three clinically prioritized comparisons, the remaining analyses were exploratory and were not adjusted for multiplicity. Residual confounding related to disease severity, study center, admission period, treatment timing, and mNGS procedures may also remain. Finally, because EBV was assessed after the development of GFAP-A, the temporal relationship between EBV detection and GFAP-directed autoimmunity could not be established.

Despite these limitations, this study provides multicenter comparative data on GFAP-A patients with and without mNGS-detectable CSF EBV. The findings suggest that CSF EBV detection is mainly associated with greater CSF pleocytosis rather than an obviously distinct MRI pattern or demonstrably worse long-term outcome. Prospective multicenter studies incorporating standardized quantitative CSF EBV PCR, comprehensive autoimmune antibody testing, serial virological assessment, and harmonized treatment protocols are needed to clarify whether EBV has a pathogenic role in GFAP-A or primarily represents a marker of concurrent intrathecal inflammation.

## Conclusion

Among the three clinically prioritized comparisons, CSF WBC count was the only variable that remained statistically significant after Bonferroni correction. This exploratory finding suggests greater CSF inflammatory activity in GFAP-A patients with mNGS-detectable CSF EBV, although definitive causal or prognostic inference cannot be drawn given the retrospective design and small sample size. The observed differences in altered consciousness and CSF chloride concentration remained inconclusive after correction for multiple testing. No statistically detectable differences were identified in GFAP-IgG characteristics, MRI findings, relapse, or functional outcomes, although clinically relevant differences could not be excluded because of the small EBV-positive subgroup. Raw EBV mNGS read counts were not statistically correlated with CSF WBC count or peak pretreatment neurological disability assessed by mRS. These findings do not establish CSF EBV positivity as an independent pathogenic or prognostic biomarker for GFAP-A, and the biological origin of the detected EBV sequences remains uncertain.

## Data Availability

The original contributions presented in the study are included in the article/Supplementary material, further inquiries can be directed to the corresponding author.
