# Supplementary material for: Clinical features associated with cerebrospinal fluid Epstein–Barr virus positivity in autoimmune GFAP astrocytopathy: a multicenter retrospective cohort study
Source: Front Neurol. 2026 Aug 27;17:1906448. doi: 10.3389/fneur.2026.1906448 (PMC13557978; doi:10.3389/fneur.2026.1906448)
Supplement: Supplementary file 1 [file Table_1.pdf]

Supplementary Table 1. GFAP-IgG Characteristics According to CSF EBV Status

Variable	CSF EBV-positive group (n = 10)	CSF EBV-negative group (n = 50)	Raw P value
Isolated CSF GFAP-IgG positivity, n (%)	6 (60.0)	32 (64.0)	1
Dual serum–CSF GFAP-IgG positivity, n (%)	4 (40.0)	18 (36.0)	1
Quantifiable CSF GFAP-IgG titer, median (IQR)	1:100 (1:32, 1:100), n = 8	1:32 (1:10, 1:100), n = 42	0.147
Qualitative TBA-only CSF GFAP-IgG positivity, n (%)	2 (20.0)	8 (16.0)	0.668

Note: Isolated CSF GFAP-IgG positivity was defined as CSF positivity with a negative serum result. Dual serum–CSF positivity was defined as GFAP-IgG positivity in both serum and CSF. Quantitative CSF GFAP-IgG titers were compared only among patients with available serial-dilution titers. Patients with qualitative TBA-only positivity without an available serial-dilution titer were excluded from the quantitative titer comparison. Categorical variables were compared using Fisher's exact test, and quantitative titers were compared using the Mann–Whitney U test. All analyses were exploratory; the reported P values are raw two-sided values and were not adjusted for multiplicity.

Abbreviations: CSF, cerebrospinal fluid; EBV, Epstein–Barr virus; GFAP-IgG, glial fibrillary acidic protein immunoglobulin G; IQR, interquartile range; TBA, tissue-based assay.

Supplementary Table 2. Individual Clinical, Laboratory, Neuroimaging, Treatment, and Outcome Characteristics of 10 Patients with Autoimmune GFAP Astrocytopathy and CSF EBV Positivity

Patient No. (sex/age, years)	Clinical phenotype	Clinical manifestations	Serum WBC ($\times10^9$/L)	Serum CRP (mg/L)	ESR (mm/h)	CSF opening pressure (mm H_2O)	CSF WBC ($\times10^6$/L)	CSF protein (g/L)	CSF glucose (mmol/L)	CSF chloride (mmol/L)	OCB type	Serum GFAP-IgG	CSF GFAP-IgG	CSF EBV mNGS raw reads	Blood EBV DNA PCR result	MRI findings	Acute-phase immunotherapy	Maintenance immunotherapy	Peak pretreatment mRS	mRS at last follow-up	Relapse
1 (F/30)	Meningoencephalomyelitis	Fever, headache, seizures, meningeal signs, limb weakness, altered consciousness, bowel/bladder dysfunction, respiratory failure	8.29	0.50	14	350	110	5.10	2.99	99	Type I	1:32	1:100	500	Negative	brain parenchymal, meninges, spinal cord, spinal leptomeninges	IVMP	Prednisone + MMF	5	4	No
2 (M/52)	Meningoencephalitis	Fever, headache, meningeal signs, limb weakness, altered consciousness, respiratory failure	6.50	0.60	20	220	147	1.00	2.50	113	Type I	1:10	1:100	340	Negative	brain parenchymal, meninges	IVMP	Prednisone	5	4	No
3 (M/47)	Meningoencephalomyeloradiculitis	Fever, headache, altered consciousness	6.48	0.80	15	130	130	1.41	2.68	117	Type I	Negative	1:32	1087	Negative	brain parenchymal, spinal leptomeninges	IVMP + IVIG	Prednisone + MMF	4	1	No
4 (M/55)	Meningoencephalomyeloradiculitis	Fever, headache, meningeal signs, limb weakness, altered consciousness, bowel/bladder dysfunction	8.63	0.50	11	210	220	1.20	2.38	119	Type I	Negative	1:100	35	Negative	brain parenchymal, meninges, spinal cord, spinal leptomeninges, periventricular radial linear enhancement	IVMP	Prednisone	5	1	No
5 (M/63)	Meningoencephalomyeloradiculitis	Fever, headache, seizures, meningeal signs, limb weakness, altered consciousness, bowel/bladder dysfunction, respiratory failure	6.75	5.96	10	240	67	0.96	2.20	104	Type I	Negative	Positive (TBA only)	550	Negative	brain parenchymal, spinal cord, spinal leptomeninges	IVMP + IVIG	Prednisone	5	3	No
6 (F/27)	Meningoencephalomyeloradiculitis	Fever, headache, meningeal signs, limb weakness, altered consciousness, bowel/bladder dysfunction	10.21	1.80	12	260	481	2.82	2.00	110	Type II	Negative	1:10	30	Negative	brain parenchymal, meninges, spinal cord, spinal leptomeninges	IVMP	Prednisone	5	1	No
7 (M/39)	Meningoencephalomyelitis	Fever, headache, meningeal signs	9.52	0.50	9	80	105	1.58	2.90	110	Type I	Negative	1:100	35	Negative	Meninges, spinal leptomeninges	IVMP + IVIG	Prednisone	2	1	No
8 (M/49)	Meningoencephalitis	Fever, headache, meningeal signs, limb weakness	5.68	0.94	6	150	33	0.96	3.00	117	Type II	Negative	1:100	33	Negative	brain parenchymal, meninges	IVMP + IVIG	Prednisone	3	2	Yes
9 (M/40)	Meningoencephalomyeloradiculitis	Fever, headache, meningeal signs, altered consciousness, limb weakness, bowel/bladder dysfunction	14.13	1.63	14	160	250	1.52	2.79	104	Type II	1:32	1:100	525	Negative	brain parenchymal, spinal cord,	IVMP + IVIG	Prednisone	4	2	No

Patient No. (sex/age, years)	Clinical phenotype	Clinical manifestations	Serum WBC (×10⁹ /L)	Serum CRP (mg/L)	ESR (mm/h)	CSF opening pressure (mm H_2O)	CSF WBC (×10⁶ /L)	CSF protein (g/L)	CSF glucose (mmol/L)	CSF chloride (mmol/L)	OCB type	Serum GFAP-IgG	CSF GFAP-IgG	CSF EBV mNGS raw reads	Blood EBV DNA PCR result	MRI findings	Acute-phase immunotherapy	Maintenance immunotherapy	Peak pretreatment mRS	mRS at last follow-up	Relapse
																spinal leptomeninges					
10 (M/59)	Meningoencephalomyeloradiculitis	Fever, headache, meningeal signs, altered consciousness, bowel/bladder dysfunction	8.62	0.50	8	300	110	1.79	2.39	116	Type I	Positive (TBA only)	Positive (TBA only)	218	Negative	brain parenchymal, spinal cord, spinal leptomeninges, periventricular radial linear enhancement	IVMP + IVIG	Prednisone + MMF	4	0	No

Note: Peak pretreatment mRS was defined as the highest mRS score recorded before the initiation of immunotherapy. GFAP-IgG results expressed as ratios represent the highest positive serial-dilution titers. “Positive (TBA only)” indicates a qualitative positive GFAP-IgG result on tissue-based assay without an available serial-dilution titer. OCB type I indicates the absence of oligoclonal bands in both serum and CSF, whereas type II indicates CSF-restricted oligoclonal bands. All 10 patients received intravenous acyclovir at 10 mg/kg every 8 h for 14–21 days, initiated within 72 h after the diagnosis of GFAP-A. CSF EBV mNGS results are presented as raw sequencing read counts and were not normalized for sequencing depth.

Abbreviations: CRP, C-reactive protein; CSF, cerebrospinal fluid; EBV, Epstein–Barr virus; ESR, erythrocyte sedimentation rate; F, female; GFAP-A, autoimmune glial fibrillary acidic protein astrocytopathy; GFAP-IgG, glial fibrillary acidic protein immunoglobulin G; IVIG, intravenous immunoglobulin; IVMP, intravenous methylprednisolone; M, male; MMF, mycophenolate mofetil; mNGS, metagenomic next-generation sequencing; mRS, modified Rankin Scale; OCB, oligoclonal band; PCR, polymerase chain reaction; TBA, tissue-based assay; WBC, white blood cell.
